# Building engagement in healthy aging outcomes research with older women with lived experience of incarceration

**DOI:** 10.1017/cts.2026.10724

**Published:** 2026-03-09

**Authors:** Sherri Anderson, Leigh Anne Covington, Shamika Davis, Amanda Emerson, Déon Cox Hayley, Catherine Johnson, Jill Peltzer, Bernard Schuster, Janet Severine, Catherine Siengsukon, Sharla Smith, Mary Taylor, Amanda Thimmesch, Alexandria Thompson, other Members of The OWLHART Network

**Affiliations:** 1 Department of Population Health, University of Kansas Medical Center, USA; 2 Older Women Leading Healthy Aging Research Together (OWLHART), USA; 3 School of Nursing, University of Kansas Medical Center, Kansas City, USA; 4 School of Medicine, University of Kansas Medical Center, USA; 5 Department of Physical Therapy, Rehabilitation Science, and Athletic Training, University of Kansas Medical Center, USA; 6 3 Quarters of the Way Done, USA

**Keywords:** Older adults, women, frailty, patient engagement, program description

## Abstract

Community-dwelling older women (age >50) with *lived experience of incarceration* (LEI) are an increasing yet mostly unacknowledged group with long-term health challenges. Research addressing aging-related health in older women with LEI is rare, and almost none engages collaboratively and equitably with older women to understand their aging-related health needs, preferences, and priorities. To foster awareness and readiness for engagement in outcomes research, we co-planned, implemented, and evaluated a sequence of interlinked activities and outputs with older women, clinicians and researchers, and community advocates. The *Older Women Leading Healthy Aging Research Together* (OWLHART) network met monthly from November 2024 to June 2025. Four to six women, two clinician-researchers, two community advocates, and a project team *shared* aging-related health beliefs, concerns, and outcome priorities; *learned* the basics of research and benefits of patient-centered outcomes research; created tools to *teach* about older women’s health after incarceration and the role of research; and developed *outreach* to disseminate the work. OWLHART models an equity-focused capacity-building approach for research engagement outside the clinical setting and with a patient group that has been overlooked as patients and as research partners.

The incarceration of older people (age >50) in US prisons and jails has risen 280% in the past 30 years [1]. Because of the accelerated aging associated with incarceration, researchers often categorize as “older adult” people with lived experience of incarceration (LEI) who are at or above age 50 [2]. When older people return to the community after incarceration, and nearly all of them do, they face many obstacles to health [3,4]. Women with LEI see higher rates and earlier onset of frailty, manage more chronic conditions, and have higher rates of premature mortality than women without LEI and more than men both without *and* with LEI [5–8]. Older women with a history of incarceration also have significantly more depression and falls [8]. In their 50s, many women still care for children (and grandchildren) as well as parents and significant others, while they struggle to meet employment and housing needs [3,9]. The health problems that older women face after incarceration reflect accumulated long-term stress from poverty; housing instability; substance use; and trauma from violence, abuse, and neglect – all of which both precede and follow from incarceration [10–13]. Meanwhile, structural barriers stemming from racism, social stigma, and ruptured family and other relationships can further complicate women’s efforts to meet basic needs that support health [11,14].

About 1.9 million women are released from U.S. jails and prisons each year [15]. Yet nearly all research on aging and incarceration and especially aging after incarceration focuses on health outcomes in men. The handful of large surveillance studies that include an emphasis on justice-involved women’s health indicate that older women with LEI are at higher risk of aging-related health disability than men with and without LEI and women without LEI. But older women with LEI are rarely the topic of research, and their distinctive needs are not addressed. In a recent literature review conducted by members of our project team [16], we located 25 studies published in 25 years (2000–2025) in which aging-related health in women with LEI – either currently or formerly incarcerated – was the focus of study. We found no interventional research, a lack also reflected in Canada et al.’s [17] systematic review of programs developed to support health of older adults in jails and prisons, where just one of the five studies located included older women.

Also missing from the literature on frailty and other aging-related health in older women with LEI was community-based participatory or other community-engaged research [16]. Community-engaged research works with patients or other groups in the processes of research to learn about a problem or situation experienced *by* the group [18]. Community-engaged methodology encompasses a spectrum of involvement, from eliciting community input on a research idea; to incorporating advisory boards for project oversight; to full partnership with organizations or patient groups to plan, conduct, interpret, and disseminate research [19]. Community-engaged methods have been credited with improving research rigor, relevance, and reach [20] and enhancing uptake of findings [21]. A community engagement methodology may be especially needful in research that addresses health in minoritized groups who have been excluded, overlooked, and even injured by researchers in the past. There is some evidence that community-based participatory research is more effective than other approaches in recruiting and retaining participants in clinical trials and developing effective behavioral interventions [22].

Much work remains to be done to effect healthier aging outcomes in women with LEI. *Older Women Leading Healthy Aging Research Together* (OWLHART) is a patient-centered outcomes research engagement capacity building project designed to create awareness, means, and opportunity for future comparative effectiveness research grounded in the needs and preferences of patients (women with LEI). The project brings together older women with LEI, clinician-researchers, and community advocates to explore, create, and plan for future research initiatives. In this communication, we describe OWLHART’s sequencing of sharing, learning, teaching, and outreach capacity-building activities in the project’s first eight months. We offer our account as an adaptable approach to fostering readiness for research engagement in groups that are unfamiliar with formal processes of research and may be excluded from research engagement. Our equity-grounded methods rest on the assumption that women with LEI bring rich, varied experiences and the desire to influence research and health care to facilitate healthier aging for themselves and other women.

## Materials and methods

### Engagement project aims and design

**Figure 1.**
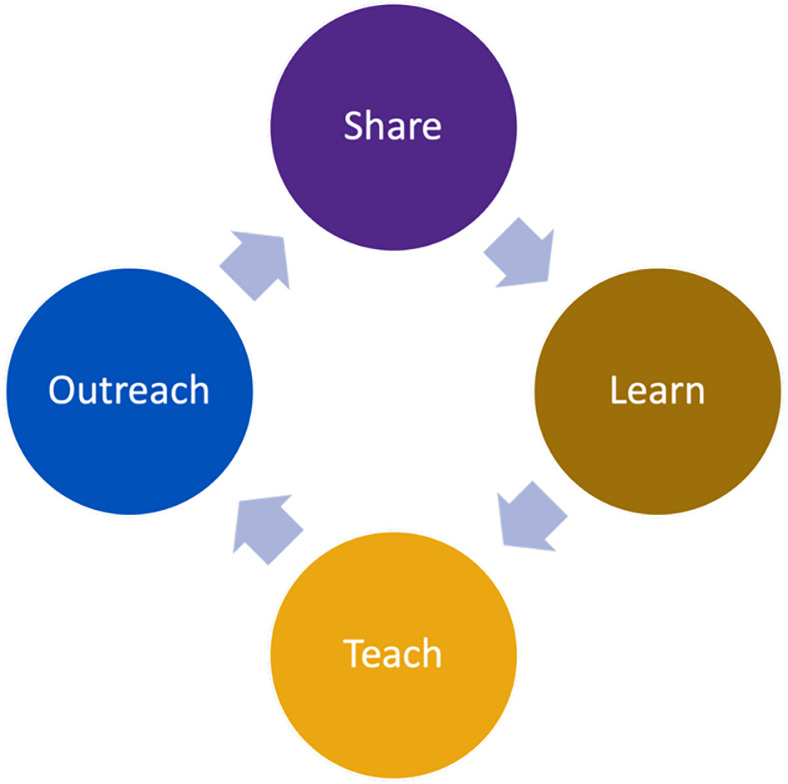
Activity domains.

OWLHART is a community-engagement capacity-building project that aims to (a) identify priority health needs and preferences of community-dwelling older women (age >50) with LEI (i.e., the “patient” group); (b) build awareness, tools, and opportunities for engagement in health care and healthy aging research with and by older women with LEI, clinician-researchers, and community advocates; and (c) set an agenda for future patient-centered outcomes research and clinical guidance with and for older women with LEI. OWLHART seeks to facilitate older women’s opportunities for meaningful participation in planning, conducting, implementing, and delivering health care and health outcomes research to address the needs that matter to them most to them. To achieve these ends, OWLHART meets monthly to share, learn, teach, and develop outreach (Figure 1) on topics related to older women’s healthy aging after incarceration. We have focused in our first eight months on building trust, learning about research, and exploring frailty and other health challenges faced by older women with LEI.

### Recruitment and compensation

OWLHART network members were recruited by member referral and word of mouth through community and previous study contacts of the project team. All network members receive $50 compensation on a debit card for each two-hour monthly meeting and $50 for acting as a member on a core team (smaller planning and working sessions). Our monthly meetings take place in the evening, when members are best able to attend, and all include dinner. The OWLHART project has also included an ongoing, embedded qualitative research study in which network members have been co-planners and participated in data (deidentified) analysis. That study was approved by the University of Kansas Medical Center IRB (#00160758).

### Activity domains – methodology

#### Sharing

OWLHART begins and ends with sharing. Sharing represents the high value the group puts on *people*, since sharing elevates women’s perspectives on frailty and other priority areas for healthy aging health and care-related preferences. Sharing means that OWLHART acknowledges power, intentionally placing women’s priorities on a level with what academic disciplines and scientific fields say is important. Sharing is about *process* because all of our activities incorporate steps that center on hearing the experiences and perspectives of women and of clinicians and community advocates and others who care for women with LEI (Table 1). Sharing is part of our monthly meeting process. Each meeting begins with a shared meal. This is a time to catch up informally on what members have been doing, hear what weighs on one another’s minds, and lift up each other’s victories and celebrations. Overall, we strive to promote equity in the group by sharing power, creating a space where all – established, new, guests, women, community advocates, team–have a voice in decision-making and leadership.

**Table 1.** OWLHART activities by domain

Activity	Domain
***A trauma-informed guide for community engaged practice*** OWLHART was divided into four groups, each starting at a different station labeled with one of the four pillars of community-engaged practice: People, Place, Process, and Power. At each station, participants called out answers to the question, “How can aspects of _[people/place/process/power] _ be handled in meetings to help me feel safe and heard?” A project team member at each station wrote responses on a sticky pad affixed to the wall, and the groups rotated after three minutes. Responses on each sheet were presented for discussion by the group. The lists guide our meeting practice and informed OWLHART’s trauma-informed engagement guide, drafts of which were reviewed and refined by OWLHART in another meeting. The guide will be shared on the website.	Sharing Teaching
***Positionality PIe: a group self-reflection resource*** We used the power wheel to guide self-reflection on the intersectional perspectives we bring (and those we are missing) as a group. Items on our modified OWLHART positionality “pie” included LEI, citizenship, gender, age, race, education, ability, sexuality, neurodiversity, mental health, housing, wealth, language, caretaking, and partnership. The project team completed the wheel separately, and then OWLHART members completed the wheel during our second meeting. OWLHART responses were collected, tallied, and reported at the following meeting in which the group discussed potential strategies to add to or compensate for perspectives that are not well-represented in our group through outreach and targeted recruitment in the listening sessions.	Sharing Teaching
***Exploring views on health and frailty (1-2–4-all activity)*** OWLHART members discussed, first as individuals (3 minutes), then in pairs (3 minutes), then in groups of four (3 minutes), and then as a large group, the prompts: “In a few words, what does health mean to us” and in a second round, “What does frailty mean to us.” Members recorded their phrases on Post-its, which the project team collected, compiled, sorted, and counted. The results were presented as frequencies and discussed at the next meeting, used in developing discussion guides for the subsequent listening sessions, as an example of content analysis in our learning about research, and as the basis for the word cloud that now serves as the OWLHART logo.	Sharing Learning
***Hearing the community: listening sessions*** OWLHART held four listening sessions in the community (*n* = 13) with three groups: older women with LEI (*n* = 7); community advocates (*n* = 4); and healthy aging researchers (*n* = 2) to explore attitudes, priorities, and perceived barriers to aging-related health and health outcomes research. Sessions were conducted on Zoom using semi-structured discussion guides based on the 1-2–4 All activity. Deidentified and collated data were analyzed by a small core team of OWLHART members and discussed by the full group. A graphic artist created an illustrated summary of each session, one of which was incorporated into a poster co-designed by OWLHART and presented by an OWLHART member and a project team member at a symposium. The graphic summaries will be shared on the project website. A co-written manuscript, in progress, reports findings.	Sharing Learning Outreach
***Fitness and frailty activity*** The *Fitness and Frailty activity* used a worksheet divided into five sections representing the five components of Fried’s [23] frailty phenotype. We set up three stations: a timed gait speed station, a grip test station, and a station in which participants responded to questions about energy, vigor, and physical activity. OWLHART members rotated through the stations, recording their data on the worksheet. The project team extracted data into a spreadsheet with a row for each “case.” OWLHART then divided again into three small groups. The groups used cut-off scores to determine a dichotomized result (frail/not-frail) for each case on a subscale worksheet. In the large group, teams called out subscale scores which were projected on a screen. We calculated a Fried frailty score for each “case” and overall and subscale scores for the whole group. We discussed how the results might be interpreted and discussed fidelity of measurement, tool reliability, self-selection bias, and small sample size.	Sharing Learning
***OWLHART patient-centered outcomes research 101 slideshow*** Project team members adapted a slide set from Godfrey et al. [24] and modified the slides’ content and appearance to be relevant, usable, and appealing to OWLHART members. The presentation used examples from the *Fitness and Frailty* activity to describe quantitative data collection, analysis, and interpretation and used elements from the *1-2–4 All* activity to illustrate qualitative processes. The slides explained how OWLHART’s listening sessions applied principles of a patient-centered outcomes research approach, and how a comparative clinical effectiveness research study might look as a next step. After discussion, we split the slides into smaller topical sets and in small groups OWLHART discussed the subsets and presented feedback to the group. The feedback informed revisions. The slide deck will be featured on the OWLHART website.	Sharing Learning Teaching Outreach

#### Learning

Learning in OWLHART includes learning what frailty is, how frailty is measured, and why it affects some groups – like women with LEI – more than others. Members’ learning also has included hands on experiences with research, to learn the basics of its purposes and process and specifically the benefits of patient-centered outcomes research. OWLHART learning is reciprocal, drawing equally on members’ expertise in their own health and the lived experiences of incarceration and transition to the community after incarceration. In learning about the science of health and frailty after incarceration, OWLHART has used a laddered, experiential process of applying quantitative and qualitative methods of data collection, data analysis, and application to learn about research (Table 1).

#### Teaching

In the teaching domain, OWLHART members are working to become instigators of future learning in others by developing tools for teaching other women with LEI and engaged community partners about frailty and resilience in women aging in the community after incarceration (Table 1). Teaching in the project also includes OWLHART’s upcoming partnership with a health communications specialist to script, act in, and film three brief informational videos on the needs and opportunities of aging-related health research by and with older women with LEI and tips for clinicians and researchers on how to approach and work respectfully with older women with LEI in community-based health care delivery and health outcomes research.

#### Outreach

OWLHART’s activities include planning the means to disseminate what we share, learn, and teach, first, through the design and launch of our project website. In planning so far, the OWLHART website aims to display the project’s aims and share OWLHART member stories; make available healthy aging research, tools, and resources (like our training videos); and publicize healthy aging events and opportunities that connect older women with LEI and their families with clinicians, researchers, and community advocates for patient-centered outcomes research and program development.

## Results

### Participants

In its first eight months, the OWLHART group has fluctuated in number. At a typical monthly meeting, attendance includes between four and six returning women with LEI, one geriatrician; one healthy aging researcher; two community advocate members; and four or five project team members (Table 2). The OWLHART women or “patient” group are 49–68 years of age, live in the Kansas City Metropolitan area (spanning Kansas and Missouri), and have at least one year’s previous incarceration but are not currently incarcerated or on supervised probation. The women’s incarceration experience ranges from one year to a couple decades’ duration.

**Table 2.** OWLHART members and project team

Member	Role	Gender	Race	Age	LEI
1	Patient	F	B	OA	Y
2	Patient	F	B	ML	Y
3	Patient	F	W	OA	Y
4	Patient	F	W	OA	Y
5	Patient	F	W	OA	Y
6	Patient	F	W	OA	Y
7	Patient	F	W	OA	Y
8	Patient	F	W	OA	Y
9	Patient	F	W	OA	Y
10	Community	F	B	OA	Y
11	Community	F	B	ML	N
12	Clinician	F	W	OA	N
13	Researcher	F	W	ML	N
14	Team	F	B	YA	N
15	Team	F	W	YA	N
16	Team	M	W	OA	N
17	Team	F	W	OA	N
18	Team	F	W	OA	N
19	Team	F	W	OA	N
20	Consultant	F	B	ML	N

*Note:* B: Black or African American; F: Female; LEI: lived experience of incarceration; M: Male; ML: midlife; OA: older adult (50 and older); W: White; YA: young adult.

Among the six regular attenders, two women are Black and four are White. The two community advocates include a Black woman who runs her own non-profit, providing a range of transition services to women as they leave prison, and a White woman who has worked with women with LEI in programming to promote cervical health. The six-member OWLHART project team includes one older adult White male and one Black woman who is also the youngest member (age 24). We have not yet recruited a member who identifies as Hispanic but have consulted with a local organization that promotes Latino health to translate our materials to attract future members from that community. Additionally, one of the community advocates has LEI, and among the patient members is a trained health care worker and a certified peer specialist.

### Activities

We summarized sharing, learning, and teaching activities from November 2024 to June 2025 in Table 1. OWLHART activities (a) include some combination of learning about aging-related health or frailty and learning about research, (b) cycle through two or more rounds of OWLHART involvement, (c) result in the creation of a tangible product that embodies the thought, time, and effort invested by the group, and (d) are scaffolded, so that any one activity informs and is informed by others that precede and follow it.

### Engagement

Members engage in OWLHART actively as planners, designers, and participants in activities. For almost all the activities in Table 1, OWLHART members were involved in brainstorming content, trying out or discussing drafts, and providing feedback as products were refined, all the while understanding that we were creating resources destined for sharing with others. When asked to select activities that mattered most to them, five OWLHART members singled out the Fitness and Frailty activity. Members appreciated *doing* the Fried [23] frailty phenotype test for the information they gained about their own physical status. Despite disclaiming that the activities were not diagnostic, one member noted appreciatively that the activity “highlights what I need to work on to not be more frail as I age,” and another said that the activity “showed me I was not as frail as I thought.” Others liked the physicality of the activity (e.g., grip test, gait speed measure), how simply doing the tests “made me use body parts I had not used in a while – made me feel healthier.”

Another valued activity was the very first, the group’s co-creation of a trauma-informed community practice guide which then guided OWLHART practice in meetings, influencing us in leaving doors open during sessions, sharing meeting agendas so members know what is coming next, and including more time for informal discussion and sharing so women know one another. Members also appreciated the activity for helping establish a sense of common ground. One member noted how the discussions helped her see “that what I’m experiencing after prison, others are going through the same thing.” Another member said it was a “comfort just to know I’m not alone.” Two members used the term “camaraderie,” and another noted how much she valued being able to “share with others and hear their stories.” Two members specified that they found activities like adapting and applying the power and positionality tool especially rewarding for “ask[ing] questions that make me think–dig deep;” “learn new things;” and hear “different perspectives.” Last, and related to communality, half the members mentioned how much it mattered to them that activities were designed to “help other women,” “help others understand older ladies that have been incarcerated,” or “make a difference in someone else’s life.” One member specified that she valued engagement in OWLHART activities because they have “purpose.”

## Discussion

Improving the healthy aging outcomes of older women with LEI is a goal that will likely be met only with significant engagement of older women who have the experience of incarceration. This is a group of women with distinctive life pathways and exposure to environments and life circumstances that increase the risk of early functional disability and chronic disease [12]. The OWLHART project was formed to involve women with LEI, clinician-researchers, and community advocates in a collaborative process of developing awareness, means, and opportunity for patient-centered outcomes research engagement around the challenges women face in achieving health in aging after incarceration. Prospectively, OWLHART aims to ready the ground for engagement at all points in the research process by older women with LEI and engaged community partners: from setting standards and priorities to co-designing interventions; creating, adapting, and implementing data collection tools; interpreting data; and evaluating and reporting findings. OWLHART goals and methods align with models like the Centers for Disease Control’s *Principles of Community Engagement* [25] and the Patient-Centered Outcomes Research Institute (PCORI) [26] updated guidance for partnerships between patients, researchers and other engaged communities, in both of which engagement spans multiple levels of involvement, from input to consultation and from collaboration to shared leadership. PCORI’s six pillars of partnership model includes representative involvement, early and ongoing engagement, dedicated funds for engagement, building capacity to work as a team, meaningful inclusion of partnership in decision-making, and ongoing review and assessment of engagement [26]. From its inception, OWLHART has integrated input, consultation, and shared decision making into the planning, activities, and deliverables of the project. The engagement-centered funding has helped ensure that members are fairly compensated for the substantial time and effort they commit to the project.

Equitable collaboration is the true heart of OWLHART and involves supporting members’ direct involvement in shaping the overall project and activities. Our collaboration rests on respect, trust, communication, transparency, and flexibility. Trust is especially necessary in the context of LEI, where deficits in carceral and transitional health care and a history of clinical research exploitation foster mistrust and distrust [27]. OWLHART activities frequently draw on *liberating structures,* mini structures of group interaction designed to promote trust through equity and inclusivity [28]. Liberating structures operate on the premise that the communities with lived experience of a need are best situated to pose the questions that matter *and* generate solutions that work. Liberating structures assume multiple sources of knowledge in a group and use flexible forms to support equity in participation. This is especially important because OWLHART comprises, in addition to the OWLHART network members, the six-member academic project team (Table 2) of faculty researchers, students, research assistants, and a community liaison with access to social and institutional power generally out of reach of most OWLHART network members. In this way, liberating structures contribute to trust and enhance capacity to work as a team in framing and meeting needs. The 1-2-4-All activity (Table 1) used to explore meanings of health and frailty among us was a liberating structure [28]. Similarly, OWLHART “core teams,” the smaller (3–4-member) teams that meet separately to plan specific activities, review products, and approve changes, provide a liberating structure within which the group extends engagement in collaboration to shared decision-making.

## Conclusion

The advantages of meaningful patient and community partner involvement in research are considerable. Maurer et al. [29] distilled five primary impacts of meaningful research engagement from interviews with investigators in 58 patient-centered outcomes research studies, and found the most frequently named impacts were research priorities and materials that better reflect patient and caregiver needs and preferences; more timely, cost-effective studies; expanded reach or compass, better study designs, and higher quality interventions; increased scope and quality of stakeholder interactions; and results that matter to patients and other stakeholders. For older women with LEI, meaningful engagement in research offers to produce better quality, more impactful outcomes, ensuring that the science is responsive to women’s needs at all points. The enthusiastic commitment to OWLHART goals and activities suggests that meaningful engagement may also function as a unique forum in which women can leverage their firsthand knowledge of incarceration and reentry, influence the science of aging and health, and act on the desire to help other women who come behind them. Such are the ripple effects that could help reestablish the public’s trust in science and reinvigorate science’s commitment to communities.
